# Blood Lead Levels and Health Problems of Lead Acid Battery Workers in Bangladesh

**DOI:** 10.1155/2014/974104

**Published:** 2014-02-25

**Authors:** Sk. Akhtar Ahmad, Manzurul Haque Khan, Salamat Khandker, A. F. M. Sarwar, Nahid Yasmin, M. H. Faruquee, Rabeya Yasmin

**Affiliations:** ^1^Department of Occupational & Environmental Health, Bangladesh University of Health Sciences, Dhaka 1216, Bangladesh; ^2^Department of Occupational & Environmental Health, National Institute of Preventive and Social Medicine, Dhaka 1212, Bangladesh; ^3^Environmental Health Unit, World Health Organization, Dhaka 1000, Bangladesh; ^4^Department of Community Dentistry, Dhaka Dental College, Dhaka 1206, Bangladesh; ^5^Addin Women's Medical College, Dhaka 1217, Bangladesh

## Abstract

*Introduction*. Use of lead acid battery (LAB) in Bangladesh has risen with sharp rise of motor vehicles. As result, manufacture of LAB is increasing. Most of the lead used by these industries comes from recycling of LAB. Workers in LAB industry are at risk of exposure lead and thus development of lead toxicity. *Objective*. The objective of this study was to measure the blood lead concentration and to assess the magnitude of health problems attributable to lead toxicity among the LAB manufacturing workers. *Methods*. A cross-sectional study was conducted among the workers of LAB manufacturing industries located in Dhaka city. *Result*. Mean blood lead level (BLL) among the workers was found to be high. They were found to be suffering from a number of illnesses attributable to lead toxicity. The common illnesses were frequent headache, numbness of the limbs, colic pain, nausea, tremor, and lead line on the gum. High BLL was also found to be related to hypertension and anemia of the workers. *Conclusion*. High BLL and illnesses attributable to lead toxicity were prevalent amongst workers of the LAB manufacturing industries, and this requires attention especially in terms of occupational hygiene and safety.

## 1. Introduction

Lead is recognized as an environmental and occupational pollutant [1–3]. Lead toxicity is one of the most prevalent occupational and environmental health problems in the world. Adults are mainly exposed to lead in their workplaces through inhalation of lead laden particulates, poor personal hygiene, and ingestion of lead-contaminated water and food which also contributes to the exposure [2–4]. Blood lead level (BLL) of 10 *μ*gm/dL was considered to be as the “level of concern” in children by CDC [5]. The US Occupational Safety and Health Administration (OSHA) lead standards require workers to be removed from lead exposure when their BLLs are equal to or greater than 50 *μ*g/dL (construction industry) or 60 *μ*g/dL (general industry) and allow workers to return to work only when the BLL is below 40 *μ*g/dL [6, 7].

Lead has toxic effects on almost all organ systems of the body [3, 4, 7–12]. Anemia is the classic manifestation of lead toxicity [4, 7–9, 13, 14]. Lead exposure reduces the lifespan of the erythrocytes and inhibition of the heme biosynthesis [4, 7, 8, 14]. “Lead line” on the gum often associated with abdominal colic, nausea, and vomiting are common gastrointestinal manifestations [2, 4, 7, 9]. Both adults and children develop neurotoxicity, children being more susceptible [2, 4, 7–9]. Diminished Intelligent quotient (IQ) level, slowness of performance, excessive sleep, and pain and tenderness in muscle have been found to increase with increasing blood lead level [2, 7, 11, 13, 15]. Extensor muscle palsy with “wrist drop” or “ankle drop” has been recognized as the classic clinical manifestation of neurotoxicity [2, 4, 8]. Chronic nephropathy, which may progress to kidney failure, is common in workers with blood lead level above 60 *μ*gm/dL [2, 4, 7, 8, 12]. Long term low level lead exposure has been associated with elevation in blood pressure [2, 4, 8, 16, 17].

In Bangladesh, use of lead acid batteries has sharply risen because of enhanced demand in the transport sector [18, 19]. About 97% lead acid batteries in Bangladesh are manufactured by recycling batteries and scrap metal. Lead recovered from old batteries by crude smelting process is used as raw material to manufacture a new battery. This recovered lead is used and subsequently recycled for several occasions [18–20]. According to Bangladesh Bureau of Statistics (BBS) baseline survey, there are 12,207 battery recycling/recharging establishments all over Bangladesh and 34% of these establishments are found in Dhaka division. Throughout the country, 22,480 persons were engaged in the battery recharging/recycling establishments and about one-fourth (24.6%) of them are child workers (5–17 years) [21]. Health of the workers in these battery recycling/recharging establishments is much neglected; there is ample chance of exposure to lead among the workers and thus the workers are at the risk of developing lead toxicity [19, 20]. In this study, an attempt was undertaken to determine the blood lead level and to assess the magnitude of the health problems attributable to lead toxicity among lead acid battery workers.

## 2. Methodology

This was a cross-sectional study, conducted among the workers of lead acid battery industries located in Dhaka city. Most of the industries were small. The workers who were working in these industries for at least one year and gave consent to participate in this study based on informed consent were selected as the respondents. A total of 118 workers from 15 selected lead acid battery industries were thus included as respondents. The respondents were interviewed and examined for health-related information by medical personnel. Subsequently, with proper precautions, 5 mL blood sample was collected from each respondent for blood lead level estimation and routine examination. Collected samples were processed and preserved for transportation to laboratory for lead and routine examination. To measure the blood lead level, stripping voltammetry technique was applied.

## 3. Results

The age of the respondents ranged from 14 to 60 years and their mean age was 31.3 ± 11.4 years. Among them, 58.5% of the respondents were less than 30 years of age and a few (6.8%) were above 50 years of age. Most (78.0%) of the respondents had education up to class-X and 18.9% of them had no education, and only 5.1% of the respondents had education above the higher secondary school certificate level. Of the total 118 respondents, 48.3% were smoker. Workers involved in opening and breaking, acidification, repair, making and sales, and other work accounted for 20.3%, 32.2%, 11.7%, 14.4%, and 21.2% of the respondents. The duration of employment of the respondents ranged from 1 to 30 years, the mean number of years in the job being 9.7 ± 7.8 years. One-third (33.9%) of the respondents had been working in these industries for 5 years or less and only 17.8% had been working for more than 15 years. The shift of work varied from 8 to 15 hours and about 28.8% of the workers worked more than 9 hours in a day. Most (56.8%) of the respondents did not use personal protective equipment while 43.2% used some kinds of PPEs at the time of work.

The mean blood lead level of the workers was found to be 65.25 ± 26.66 *μ*gm/dL.  Table 1 shows the blood lead concentration of the respondents according to their working sections. Workers involved in acidifying were found to have high blood lead level (78.70 *μ*gm/dL), followed by those involved in plate making process (73.57 *μ*gm/dL) and opening and breaking of old batteries (66.77 *μ*gm/dL). On the other hand, lead level was found to be less (39.70 *μ*gm/dL) among the workers not involved in battery breaking or manufacturing process (administration and security personnel).

Table 2 shows the mean blood lead levels of respondents by years of employment, duration of daily shift, PPE use, and personal habits. The mean blood lead level of the workers varied with duration of shift; workers who worked more than 8 hours in a day had statistically higher (*t* = 2.209; *P* = 0.029) mean blood lead level (70.22 ± 30.15 *μ*gm/dL) compared to that of workers (59.56 *μ*gm/dL) who worked up to 8 hours. It was found that the workers who smoked had higher mean blood lead level (71.50 *μ*gm/dL) than nonsmokers (59.35 *μ*gm/dL), and the difference was statistically significant (*t* = 2.554; *P* = 0.012). The workers who had habit of bathing regularly after completion of daily work had significantly (*t* = −2.81; *P* = 0.008) lower blood lead level (55.97 *μ*gm/dL) compared to the workers who did not bath regularly (69.66 *μ*gm/dL).

Table 3 shows the illnesses attributable to lead toxicity that had developed amongst the respondents after they had been employed in the lead acid battery industry. Those having headache, numbness, colic pain, nausea, tremor, lead line on the gum had significantly (*P* < 0.05) higher mean BLL than those who did not have such illnesses.

Table 4 shows the haemoglobin (Hb) level of the respondents ranged from 9.75 to 13.50 gm/dL and the mean Hb level was 11.40 (±0.747) gm/dL. Out of the total 118 examined blood samples, 33 (28.0%) respondents were found to be anaemic and of them 49.1% had microcytic anemia, 30.4% had normocytic anaemia, and 21.1% had macrocytic anaemia. Three blood samples showed red blood cell with basophilic stippling. Table 4 shows that respondents having lower Hb levels had higher mean BLL. Moreover, those who were anaemic had significantly (*P* < 0.001) higher mean BLL (81.83 ± 26.27) than those who were not anaemic (58.81 ± 23.89).

Table 5 shows the blood pressure and mean blood lead level of the respondents. The blood pressure of the workers was categorized into normotensive (diastolic BP ≤ 90 mm Hg and systolic BP < 140 mm Hg) and hypertensive (diastolic BP > 90 mm Hg and systolic BP ≥ 140 mm Hg). Of the total respondents, 29.8% were found to be hypertensive. Workers having hypertension were found to have significantly (*P* = 0.027) higher BLL (73.70 ± 30.03 *μ*gm/dL) than those who were normotensive (61.83 ± 24.40 *μ*gm/dL).

Multiple regression analyses (primarily enter method and then stepwise method) showed that the exposure category, duration of work shift, and regular bathing at end of the work day together could explain 35.30% (Table 6) of the variance of blood lead concentration (*F* = 22.292, *P* < 0.001). In the final model, the exposure status had a strong explanatory capacity and was demonstrated as a good predictor and alone was accounted for 24.4% of the variance of blood lead concentration (*P* < 0.001). Its change by one standard deviation, while holding duration of work shift and regular bathing at end of the work day as constant, would change the blood lead concentration by 0.425 standard deviations.

## 4. Discussion

Most of the lead acid battery industries are cottage industry and situated in the thickly populated residential areas of old Dhaka city. These industries had small premises; many of the operations are carried out in open air and the melting operation to recover lead from scrap is usually carried out in small rooms having almost no exhaust fan or chimney. The workers mentioned that the working room was too hot and complained about poor ventilation and lighting. They also mentioned that less working space and roadside situation of the factories made them work with difficulties. The workers were found to take their food in the same working room.

The respondents in the current study were mostly (60%) young (≤30 years of age). About 19% of the workers were illiterate and another 28% had undergone five years of schooling. Considering the present age of the workers and their years of job, most of the workers were exposed to lead from young age and continuing for long time. It is well reported that the children are most vulnerable to lead; they absorb more lead than adults and they are more susceptible to develop lead toxicity, particularly neurological toxicity even at low level exposure [2, 7, 8, 15].

Almost half (48.3%) of the workers of this study were smokers and were found to smoke during work with contaminated hand; they did not wash their hand before smoking. On the other hand, during smoking in work place, the cigarette gets contaminated by settling of air borne lead dust and fumes on it, and also due to increasing hand to mouth movement, the workers might ingest more lead due to smoking [22, 23]. In this current study, the BLL was found 12.22 *μ*gm/dL higher among smokers compared to that of nonsmokers (*P* = 0.012). This finding is consistent with other study findings, which reveal that smoking at workplace is significantly related to blood lead concentration and BLL is found to be higher among the smokers than nonsmokers [22–24].

Most of the respondents had high BLLs, and the mean BLL was 65.25 *μ*gm/dL; BLL of 84% of the respondents was found to be >40 *μ*gm/dL whereas about 50% of the respondents had mean BLLs of 60 *μ*gm/dL or more. According to OSHA, the blood lead level should be below 40 *μ*gm/dL and if it is more than 40 *μ*gm/dL, the worker must be notified in writing and provided with medical examination [5, 6]. Further, if the workers have blood lead level of 60 *μ*gm/dL for a single time or blood lead level over the past 6 months exceeded 50 *μ*gm/dL, the worker must be removed from his job and can be placed to a job of lower exposure. The workers may return to the job if two consecutive blood lead levels are less than 40 *μ*gm/dL [6, 7]. Therefore, it could be apprehended that the workers of these industries were chronically exposed to lead and were at the risk of developing lead toxicity.

In this study, higher BLL was found among the workers who are working in opening and breaking of old lead battery, melting recovered lead, casting and molding lead plates, trimming plates, applying lead oxides to the plates, and forming, acidification, and assembling of new battery. The workers of these sections were at high risk of lead exposure because of the possibility of coming in direct contact with lead during handling of recovered lead, making and trimming new lead plates, pasting lead oxides, and finishing of new lead battery. Inhalation of lead might also occur by air borne lead particulate matter and by fumes during melting to recover lead [24–27]. Further, lead exposure may occur through ingestion of contaminated food and drink and, in this study, as the workers took their food and drink in the premises. The personnel engaged in administration and security were also found to have some lead (39.70 *μ*gm/dL) in their blood, but this was significantly lower than levels found in blood of workers who were involved in the recycling of lead from used batteries and manufacture of batteries from recovered lead.

In the current study, with the variations of years of job, no significant difference of blood lead level was found. However, with the increased daily working hours (shift of work), there was significant increase of BLL. On the other hand, after adjustment for various confounding variables, strongly significant association was found between exposure status and blood lead level. Multivariate regression analysis demonstrated that exposure category had a strong explanatory capacity and was identified as strong predictor (*R* ^2^ = 24.4%) for blood lead concentration. The similar type of findings was obtained from an Indian study on lead, where it was mentioned that mean blood lead level was significantly more in the high exposed section compared to low exposed section of the lead factory [27].

Chronic exposure to lead may cause toxicity which affects gastrointestinal, hematopoietic, nervous, renal, and reproductive system and may cause occurrence of various diseases [2–4, 7–13, 15]. In this study, a number of manifestations which are attributable to chronic lead toxicity, for example, weakness (38.7%), frequent headache (34.7%), pain in the limbs (34.5%), weakness of the limbs (33.1%), fatigue (27.7%), and numbness (25.7%), were prevalent among the workers. Prevalence of above illnesses was related to high blood lead level (more than 60 *μ*gm/dL) and in a number of illnesses, the blood lead level of the workers was significantly high, for example, frequent headache (*P* = 0.041), numbness of the limbs (*P* = 0.044), colic pain (*P* = 0.008), nausea (*P* = 0.045), tremor (*P* = 0.011), and lead line on the gum (*P* = 0.034).

Chronic lead toxicity has effect on hematopoietic system and presence of anaemia indicates significantly elevated blood lead for prolonged period [4, 7–9, 13, 14]. This anaemia results due to impairment of haem synthesis and acceleration of red blood destruction [4, 7, 8, 14]. The types of anaemia which commonly occur in lead toxicity are normocytic normochromic and microcytic hypochromic types [4, 7, 14] and in this study also normocytic, microcytic, and macrocytic anemia were found. Moreover, it was found that the mean BLL was significantly (*P* < 0.001) high among the workers who had anaemia (81.83 *μ*gm/dL) compared to that of nonanaemic (58.81 *μ*gm/dL) workers. In addition to anemia, basophilic stippling of erythrocytes was also found in three cases, which is also an indication of significant intoxication of lead for prolonged period [4, 14].

Prolonged and high level of lead exposure particularly of occupational exposure were reported to cause elevated blood pressure [2, 4, 8, 16, 17]. Some studies showed that prolonged environmental and occupational exposure even to low level of lead can be associated with the occurrence of elevated blood pressure [8, 28]. However, about 30% of the respondents of this study were found to have been suffering from hypertension and the hypertension was observed amongst those who had significantly (*P* = 0.027) higher blood lead level (73.70 ± 30.03 *μ*gm/dL).

## 5. Conclusion

Lead used in recycled lead acid battery industries come from lead recovered from old or used batteries. The process involved in recovering lead is crude, and is the most likely cause of unusually high exposure to lead. In the present study, it is evident that the workers working in lead acid battery industries had high level of lead in blood. And they are found to be suffering from many illnesses attributable to lead toxicity. Furthermore, the poor working environment, inappropriate use of PPEs, and long work shift could be considered as influencing factors.

## 6. Recommendations

The handling of lead during different processes of lead acid battery manufacturing should be done in such way that the lead exposure is minimum to the workers. The workers should be provided with proper working environment like sufficient working space and proper ventilation. Measures should be taken to restrict or control any source of lead dust or fumes by applying proper technical control measures in every step of lead acid battery manufacturing process. To limit the exposure, the workers who had long years of job could be shifted to the less exposed section. Workers should be made aware to undertake precautionary measures to use PPEs and maintain personal hygiene. Periodic estimation of blood lead level and examination of manifestations attributable to lead toxicity are to be undertaken for early detection and for preventive measures as well.

## Figures and Tables

**Table 1:** Blood lead level of respondents according to working sections.

Working section	Frequency	Mean *μ*gm/dL	Std. deviation
Opening and breaking	24	66.77	26.29
Acidification	38	78.70	27.11
Repair	14	61.70	17.40
Making	17	73.57	23.81
Administration and other	25	39.70	11.36

**Table 2:** Blood lead level of respondents according to years of job, daily working hour, and personal habits.

Factors	Frequency	Mean *μ*gm/dL	Std. deviation
(1) Years of job			
Up to 5 years	40	61.05	20.81
>5–10 years	30	73.29	31.26
10.1–15 years	27	60.50	24.83
Above 15 years	21	67.87	29.95
(2) Shift of work in hour			
8	55	59.82	20.20
9	29	66.00	29.35
10	15	69.36	24.17
11	13	69.91	32.68
12	4	81.10	33.16
15	2	111.05	69.22
(3) PPE use			
Yes	51	61.95*	23.93
No	67	67.76*	28.34
(4) Smoking habit			
Yes	57	71.50**	29.40
No	61	59.35**	22.29
(5) Bath regularly after daily work			
Yes	38	55.97***	24.24
No	80	69.66***	26.57

*t = 1.178; *P* = 0.241, **t = 2.554; *P* = 0.012; ***t = −2.81; *P* = 0.008.

**Table 3:** Illnesses attributable to lead toxicity and mean blood lead level of workers.

Illnesses attributable to lead toxicity	*n* = 118	Mean BLL (*μ*gm/dL)	Std. dev.	*P* value
Headache				
Yes	34 (28.8%)	73.09	31.26	*t* = 2.089
No	84 (71.2%)	62.08	23.90	*P* = 0.039
Numbness of limbs				
Yes	31 (26.3%)	73.47	34.32	*t* = 2.032
No	87 (73.7%)	62.32	22.73	*P* = 0.044
Colic pain				
Yes	20 (16.9%)	79.50	36.09	*t* = 2.700
No	98 (83.1%)	62.34	23.37	*P* = 0.008
Nausea				
Yes	13 (11.0%)	79.14	31.89	*t* = 2.024
No	105 (89.0%)	63.53	25.49	*P* = 0.045
Tremor				
Yes	25 (21.9%)	77.20	28.66	*t* = 2.593
No	93 (78.1%)	62.04	25.19	*P* = 0.011
Lead line on gum				
Yes	33 (28.0%)	73.54	26.25	*t* = 2.143
No	85 (72.0%)	62.01	26.14	*P* = 0.034
Weakness				
Yes	47 (39.8%)	68.29	30.66	*t* = 1.011
No	71 (60.2%)	63.24	23.49	*P* = 0.314
Fatigue				
Yes	30 (25.4%)	70.48	30.74	*t* = 1.250
No	88 (74.6%)	63.47	24.94	*P* = 0.274
Constipation				
Yes	29 (24.6%)	71.45	33.64	*t* = 1.454
No	89 (75.4%)	63.23	23.70	*P* = 0.149
Anorexia				
Yes	26 (22.0%)	72.72	33.18	*t* = 1.635
No	92 (78.0%)	63.14	24.18	*P* = 0.105
Pain Limb				
Yes	38 (32.2%)	70.97	30.16	*t* = 1.621
No	80 (67.8%)	62.54	24.43	*P* = 0.108
Disturbed sleep				
Yes	29 (24.6%)	71.34	30.97	*t* = 1.427
No	89 (75.4%)	63.27	24.65	*P* = 0.156
Drowsiness				
Yes	22 (18.6%)	70.85	28.71	*t* = 1.097
No	96 (81.4%)	65.97	26.05	*P* = 0.275

**Table 4:** Mean BLL, haemoglobin (Hb) level, and anaemic status of the workers.

Factors	Frequency *n* = 118	Mean BLL (*μ*gm/dL)	Std. deviation
(1) Hb (gm/dL)			
Less than 11.00	33	81.83	26.27
11.00–11.99	54	60.31	26.24
12.00–12.99	24	59.96	18.64
13.00+	07	44.04	17.24
(2) Anaemia			
(a) Anaemic*	33 (28.0%)	81.83	26.27
Normochromic	10 (30.3%)	83.31	35.39
Microcytic	16 (48.5%)	79.41	24.71
Macrocytic	07 (21.1%)	85.27	15.54
(b) Not anaemic*	85 (72.0%)	58.81	23.89

*t = 4.566; *P* < 0.001.

**Table 5:** Blood pressure status and mean blood lead level of the respondents.

Blood pressure	Frequency (%)	Mean blood lead (*μ*gm/dL)	Std. deviation	*P* value
Normotensive	84 (71.2%)	61.83	24.40	*t* = −2.235 *P* = 0.027
Hypertensive	34 (29.8%)	73.70	30.03	
Total	**118 **	**65.57 **	**26.57 **	

**Table 6:** Predictions of blood lead concentration.

Variables in the model	Blood lead concentration					Constant
	*R* ^2^ (%)	*F*	*P*	Beta coefficient	
				Unstandardized	Standardized
Exposure category	24.4	38.806	<0.001	−32.425	−0.501	104.549

Duration of work shift	7.1	9.915	0.002	5.329	0.281	16.843

Regular bathing at end of the day	5.0	7.190	0.008	−13.682	−0.242	83.344

Exposure category + duration of work shift	27.9	23.588	<0.001	−30.246	−0.467	66.829

Exposure category + regular bathing at end of the day	27.8	23.518	<0.001	3.861	0.203	118.150
				−31.306	−0.483	

Duration of work shift + regular bathing at end of the day	18.4	14.199	<0.001	−11.314	−0.200	24.645
				7.464	0.393	

Exposure category + duration of work shift + regular bathing at end of the day	35.3	22.292	<0.001	−20.572	−0.363	68.72
				−27.508	−0.425	
				5.749	0.303	
				−16.907	−0.299	
